# Robust Ulcer Classification: Contrast and Illumination Invariant Approach

**DOI:** 10.3390/diagnostics12122898

**Published:** 2022-11-22

**Authors:** Mousa Alhajlah

**Affiliations:** Computer Science and Information Systems Department, Applied Computer Science College, King Saud University, Riyadh 12571, Saudi Arabia; mhajlah@ksu.edu.sa

**Keywords:** gastrointestinal disease, deep learning, contrast enhancement, ulcer disease classification, data augmentation

## Abstract

Gastrointestinal (GI) disease cases are on the rise throughout the world. Ulcers, being the most common type of GI disease, if left untreated, can cause internal bleeding resulting in anemia and bloody vomiting. Early detection and classification of different types of ulcers can reduce the death rate and severity of the disease. Manual detection and classification of ulcers are tedious and error-prone. This calls for automated systems based on computer vision techniques to detect and classify ulcers in images and video data. A major challenge in accurate detection and classification is dealing with the similarity among classes and the poor quality of input images. Improper contrast and illumination reduce the anticipated classification accuracy. In this paper, contrast and illumination invariance was achieved by utilizing log transformation and power law transformation. Optimal values of the parameters for both these techniques were achieved and combined to obtain the fused image dataset. Augmentation was used to handle overfitting and classification was performed using the lightweight and efficient deep learning model MobilNetv2. Experiments were conducted on the KVASIR dataset to assess the efficacy of the proposed approach. An accuracy of 96.71% was achieved, which is a considerable improvement over the state-of-the-art techniques.

## 1. Introduction

The ulcer has become a proliferating disease and can be death-causing if left untreated and may become cancerous. Helicobacter pylori bacteria or non-steroidal anti-inflammatory drugs (NSAIDs) are considered to be one of the major causes. However, the increasing use of medicines, drugs, and low-quality food has become a major health problem for the world community [1]. Physical tests for ulcers are usually expensive and some tests such as endoscopy can be painful so automated and early detection can be helpful for the doctors to cure and prevent this most common disease. The stomach lining is protected by a thick layer of mucus and serves as defense against acids and digestive juices. The esophagus, jejunum, stomach, and duodenum are mainly affected by ulcers. If left untreated, these ulcers become chronic and may cause cancer [2] in a few circumstances.

With advancements in computing and graphics, computer vision has greatly improved. It helps in solving may problems, especially in the field of medical imaging for breast cancer, brain tumors, ulcers, and many others. Automated early detection and classification can improve the accurate diagnosis and treatment. Wireless Capsule Endoscopy (WCE) [3] is not painful and can be used for visualization and detection. Doctors and practitioner’s time can be saved by not being involved in examination procedures, which is a time-consuming process.

**Figure 1:**
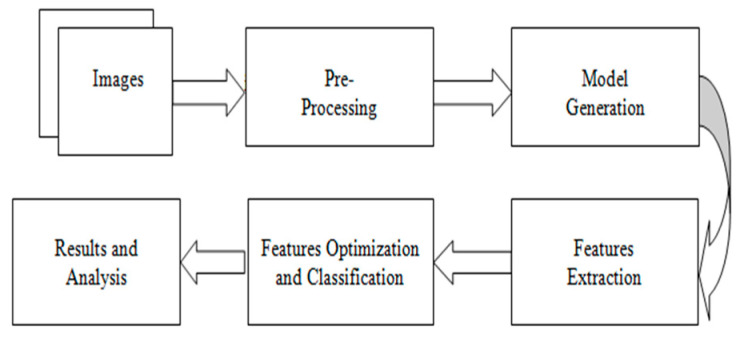
Generic automated classification flow diagram.

Preprocessing, feature extraction, and classification is a traditional computer vision task. Preprocessing such as contrast enhancement and illumination improvement can improve the overall learning task and improved performance can be achieved. Relevant feature extraction plays a significant ulcer classification from WCE images. Figure 1 shows the generic procedure for automated classification.

Deep learning techniques have outperformed most of the traditional techniques in terms of significant features and classification [4,5]. These techniques rely on large data and robust features which are extracted handle overfitting better than most of the traditional techniques. Numerous convolutional neural network (CNN) models have been developed [6,7,8,9,10]. However, it has been observed that the previous models lacked the core steps of preprocessing, i.e., contrast enhancement, and illumination balance. Models presented in the literature have been found to utilize more features, and there is also room for improvement in accuracy. Considering these research gaps, this article proposes a model with the following major contributions:

▪ The development of contrast enhancement and illumination invariance technique by the fusion of two techniques, namely, log transformation and power law transformation.
▪ The utilization of deep transfer learning for efficient feature extraction from an intermediate convolutional layer using a lightweight deep learning model, i.e., MobilNetv2.
▪ The selection and optimization of significant features utilizing PCA to improve classification accuracy using cubic-SVM.

The organization of the remaining paper is as follows. The proposed methodology is described in Section 3. Experimental results along with their discussion and comparison with existing techniques are presented in Section 4. Finally, Section 5 concludes this work.

## 2. Related Work

In the literature, the ulcer detection methods using WCE images are divided into two categories which are based on features extraction: handcrafted-feature-based methods and deep-learning-based methods. As handcrafted feature approaches encode just a portion of the information in WCE photos, many researchers choose to employ deep learning methods to detect ulcers in WCE images. For ulcer identification and categorization, Alaskar et al. [4] employed deep learning networks. To categorize WCE photos as ulcerous or non-ulcerous, they used two deep learning architectures: AlexNet and GoogLeNet. Using WCE video frames, they created experimental image datasets. In a separate study [5], the researchers used 6-layer CNN to detect ulcers. To select the region of interest, statistical color features were extracted first, followed by thresholding. Following the thresholding phase, the found region of interest is fed into a 6-layer CNN model. The final stage involves generating entropy-based characteristics for the final layer, which are subsequently presented to the classifier for classification. An average accuracy rate of 96.4% was obtained after completing research on privately provided datasets. V and Prashanth [6] also present a deep CNN for detecting ulcers at different ratios. They used 1000 to 10,000 WCE images of ulcers and non-ulcers in their research. The researchers also looked into different network depths and node configurations. The performance of the 3 × 3 convolution filter was reported to be satisfactory. In addition, they examined hyper-parameters such as drop schemes, activation functions, optimizer, number of layers, learning rate, pooling schemes, and epochs during their experiments.

Another paper [7] describes a novel technique for classifying and detecting gastrointestinal illnesses. To improve the lesion contrast, the researchers applied HSV transformation, 3D-median filtering, and 3D-box filtering in the pre-processing step. Second, a binary image was constructed and used in the saturated channel after geometric characteristics were extracted. To extract aspects such as waves, form, and color, binary segmentation and HSV pictures were used. Subset feature selection was based on correlation coefficient and principal component analysis. For categorization, a support vector machine was utilized. WCE images were analyzed by Wang et al. [8] to improve the CNN-based architecture and identify ulcers effectively. For ulcer detection, they looked at deep learning architectures, and then proposed a HANet architecture. ResNet-34 was the foundation for the fused hyper features employed in their final diagnostic judgement. They identified ulcers with a success rate of 92.05%.

Various researchers have used many different traditional machine learning techniques for predicting the different types of peptic ulcer. Grace Lai, Hung Wong et al. [10] developed a peptic ulcer machine learning model which was built on a retrospective cohort of 22,854 patients (training cohort) diagnosed with peptic ulcer disease in 2007 and 2016. He used logistic regression and ridge regression and obtained a maximum accuracy of 82.6% and 83.3%. Sen Wang et al. [8] proposed a Second Glance framework for ulcer detection and verified its efficiency and strength on a large scale WCE (Wireless Capsule Endoscopy) images dataset which consisted of 1504 independent WCE videos. The performance of his method/framework achieved the best ROC-AUC, which was 0.9235. Khan et al. [11] presented a fully automated system for the diagnosis of gastric infection. The diagnosis of gastric infection was carried out by numerous types of features, extraction features, fusion features, and robust features selection. The main problem in the GI tract diseases was that there were many similarities in the pattern of the infected region. So, Khan introduced a modern CAD which contained handcrafted features, fusion features, and deep CNN features. Datasets used in this paper were from Kvasir, CVC-ClinicDB, Private, and ETIS- LaribPolypDB. The accuracy they achieved by using this dataset was 96.5%. Ouiem Bchir et al. [12] compared the performance of different visual descriptors for the detection of ulcers using WCE (Wireless Capsule Endoscopy) frames. This comparison was intended to determine which visual descriptor represents better WCE frames and yields more accurate gastrointestinal ulcer detection. During the experiments, different visual descriptors were used along with SVM classifiers. The authors achieved maximum accuracy of 98.85% using an LBP descriptor along with SVM classifier. Jinn-Yi Yeh et al. [13] employed a technique which utilized a WCE images dataset for the detection of bleeding and ulcers and used color features. They combined all the features of images in one matrix and also engaged texture information. This matrix of features was passed to different classifiers such as support vector machine, neural network NN), and decision trees. Different performance measures were incorporated for evaluation and achieved an accuracy of 92.86–93.64%, respectively. They try to resolve the problems of low contrast images and variation in the shape of the lesion. Authors in [14] tried to resolve these problems by proposing a novel automated method for WCE images classification which involves color features and color coherence vector for determining the small intestine status and classifying it using the support vector machine. They implemented a novel method for the automated detection and classification of gastrointestinal infection. By employing the proposed automated method, the authors achieved a maximum accuracy of 98.3%. Rashid et al. [15] projected a computer aided methodology for the accurate diagnoses of stomach diseases from WCE pictures. The presented system had four basic steps, i.e., before implementing active contour segmentation, color transformation was performed using HSI (hue, saturation, intensity) color transformation. Then, the saliency-based method is implemented in the YIQ color space. After that, image fusion was performed and the last step was using SVD, LBP, and GLCM, where the extracted features were fused. In the end, the classification was carried out using neural networks. The dataset contains 9000 ulcer, bleeding, and healthy samples.

From the above discussion, it has been observed that the previous models lacked core steps of preprocessing, i.e., contrast enhancement, and illumination balance. Models presented in the literature have been found to utilize more features and there is also room for improvement in accuracy.

## 3. Material and Methods

**Figure 2:**
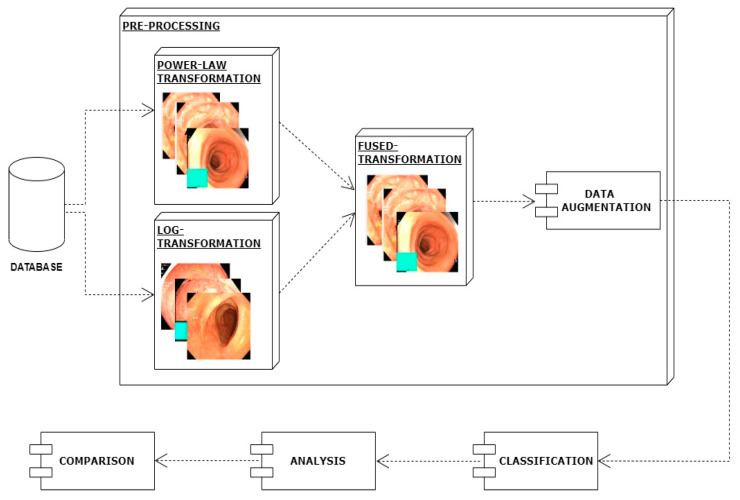
Proposed system architecture.

In this work, I have proposed a method for the classification of gastrointestinal diseases. The first step of the proposed method is preprocessing. In the preprocessing step, images picked from the dataset are enhanced by applying the log transformation technique and in parallel power law transformation. Then, both versions of enhanced images are combined which further improves images in terms of contrast and achieves better classification results. In another preprocessing step, data augmentation is applied using translation, rotation, cropping, and flip. Then I employed a deep learning technique, i.e., mobilenetv2 for the efficient classification of different types of gastrointestinal diseases. The architecture of the proposed model is shown in Figure 2.

## 4. Preprocessing

### 4.1. Power Law Transformation

**Figure 3:**
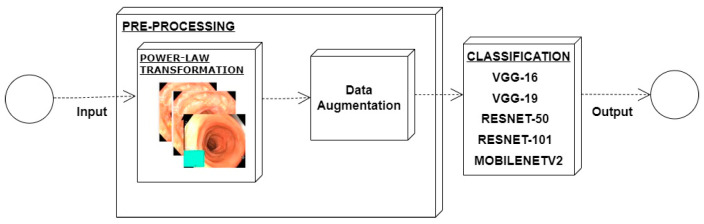
System architecture after applying power law transformation.

In an image which is poorly contrasted, the detection of an object is difficult because of its form. As it is evident from research that power law transformation enhances the images contrast [3], I applied it on our dataset as shown in Figure 3. It can be visually observed that there is clear improvement in the contrast of the image dataset.

The basic formula for the power law transformation is given below:

(1) $$\alpha =CP^{\beta }\;$$

where *C* is constant, *P* is the value of original pixel of an image and *β* is a value which needs to change to achieve better transformation. I have applied different values *C* of *β* and take the value on which better results are achieved. For the value of *C* when used above 1, it is observed that better enhancement is being achieved; moreover, when the value of *β* is closer to 1, the better contrast was observed. Table 1 shows different results for different values of *C* and *β.* It is evident from the table that the best results were achieved when I have set the value of *C* equal to 1.5 and the value of *P* equals 0.9.

### 4.2. Log Transformation

**Figure 4:**
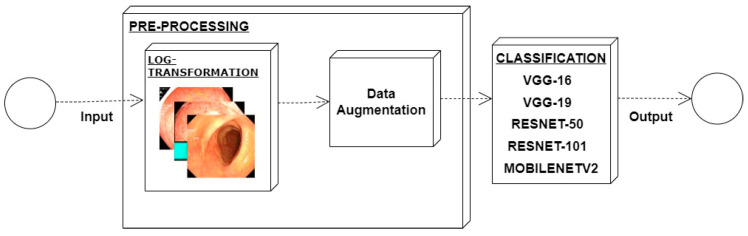
System architecture after applying log transformation.

Low contrast images have poor visual appearance and it becomes difficult to extract or detect region of interest. Contrast enhancement can improve the automated system efficiency. Log transformation expands the dark pixels’ values and compresses the high-level values [4]. As evident from the research, log transformation enhances the images with poor illuminations [5]. I applied it to our dataset as shown in Figure 4.

The results shown in the Table 2 are with the best values when log transformation is applied, which is *C* equal to 2. The illumination issue was resolved because of log transformation and the contrast issue was resolved by power law transformation.

The basic formula for the log transformation is given below:

(2) α=C Log (P+1) 

In the equation, *C* is a constant whose value can be changed and different variations of values can be applied to achieve better enhancement. I have applied different values of *C* and it is observed that when the value of *C* is closer to 2, better enhancement was achieved. It is evident from the table that the best results were achieved when I have set the value of *C* equals to 2.

**Figure 5:**
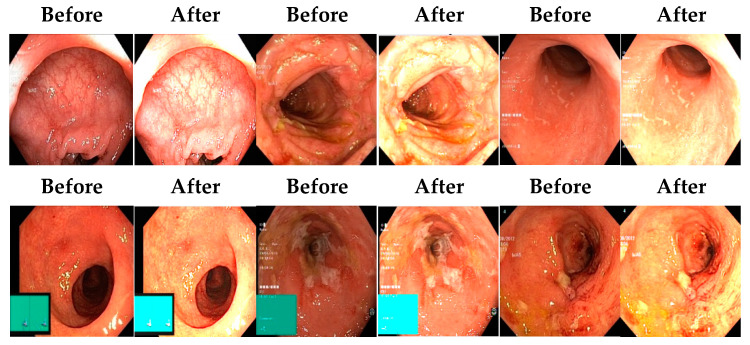
Results after fusing both transformations.

Afterward, I have fused images in which the better enhancement is received from power law, *C* equal to 1.5 and the value of *P* equals 0.9, and for log transformation, *C* equals 2. Different samples of images before and after complete enhancement are shown in Figure 5.

After applying enhancement on a complete dataset, augmentation was applied using different transformations.

### 4.3. Augmentation

**Figure 6:**
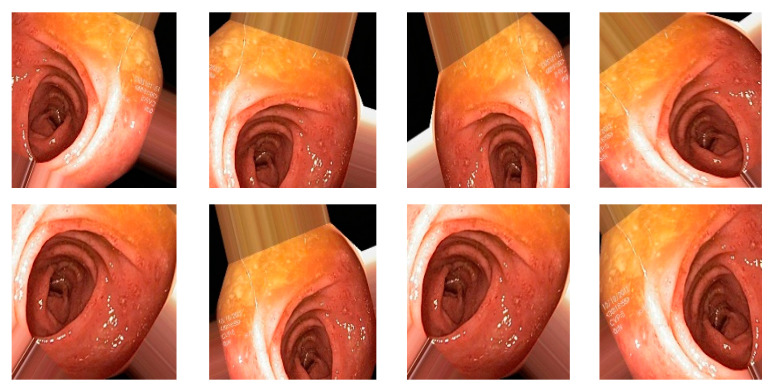
Augmented image samples.

Data augmentation is a technique to increase the size of dataset by applying different transformations on the images such as rotations, scaling, translation, flipping, and zooming [6], and is also used to avoid overfitting. For our dataset, I have applied transformations by setting the following values on each transformation: shear range value set to 0.2, zoom range set to 0.2, horizontal flip set to true, and rescale value set to 1 [7]. Total number of original images is 4000 (8 classes and 500 image for each class), and after applying the augmentation dataset, it increased to 20,000 (8 classes and 2500 image for each class). When augmentation was applied, the samples of images are shown in Figure 6.

### 4.4. Feature Extraction Using Deep Learning Method

For a model generation, different convolutional neural network models were used for the extraction of features such as ResNet-50, ResNet-101, VGG16, VGG19, and Mobilenetv2. When these models were applied and obtained results in term of accuracy, precision, and recall, it was observed that MobileNetv2 provides us with the best results and this is the reason why I used it for our proposed model. Numerical results of performed experiments are shown in Section 4.

### 4.5. MobileNetV2

**Figure 7:**
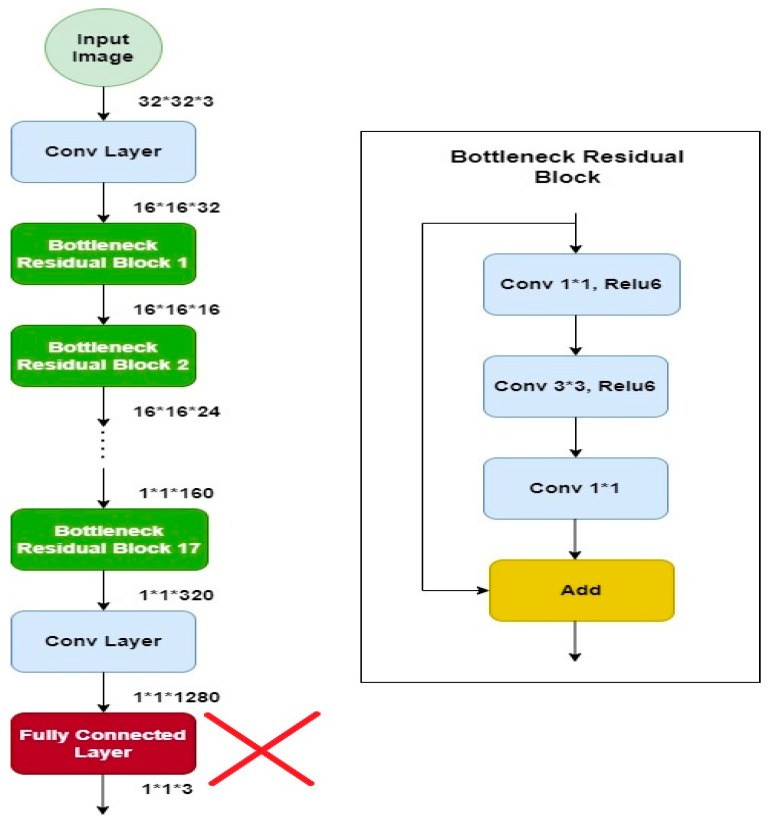
Mobilenetv2 architecture.

MobileNetv2 is a well-organized and effective convolutional neural network, specifically developed for bearing in mind the prerequisites of portable devices which have tight constraints of resources. It retains a slightly better accuracy for some datasets than other models but significantly reduces the need for resources. It is based on a reversed residual architecture in which the connections are among the bottleneck layers. It has the same architecture such as convolutional neural network but with one major difference: instead of having a full convolutional operator, it works with the depth wise separable convolutions. This is the basis of an efficient architecture of neural network [8]. This model has shown its efficiency in terms of time as well as accuracy, therefore it is used for solving many classification problems [9]. The architecture of the model is shown in Figure 7 [10]. The architecture contains the convolution layer with 32 ∗ 32 filters, after the 19 residual bottleneck layers are stacked. Filter size of 3 × 3 is used as it is considered as standard for modern networks, and dropout and batch normalization are employed for training. It uses 3.4 million parameters and is computationally less expensive.

## 5. Experimental Results and Discussions

For the experimental evaluation of the proposed model, Jupyter Notebook v6 4.11 along with libraries Keras, Pandas, Numpy, and CV2 were used. The settings used for running the different models were as follows: value of batch size is set to 32, and Adam optimizer was used. Accuracy, precision, and recall were the metrics used for the evaluation of results using the proposed methodology. CNN models were run and the stopping criteria for epochs was set to be 150 or maximum accuracy, i.e., 100%. Experimental results presented below are shown using 70:30 training to test ratios.

Firstly, I performed experiments on the dataset before processing. Results are shown in Table 3 with accuracy, precision, and recall. Well-known CNN models were tested using 70% data for the training and 30% for the testing. A maximum accuracy of 58.82% was achieved using MobilenetV2. All the other models showed lower accuracy and other performance metrics.

Power law transformation was applied to enhance the contrast of the image dataset using Equation (1). As discussed in preprocessing section, different values of *P* and *C* were tested and the best results were obtained using *C* = 1.5 and *P* = 0.9. Classification was performed using same CNN models and results are presented in Table 4. There is clear improvement in classification performance of all the classification methods. Again, MobileNetv2 performed better than all the other classifiers with an accuracy of 73.7. Additionally, there was improvement in precision from 57.32 to 83.78 and recall from 59.35 to 79.87.

Experiments were conducted by applying log transformation on the dataset. Different values were tested for image illumination and contrast enhancement, and the optimal value was set for the purpose of classification. A value of is *C* was set to 2. Classification accuracy, precision, and recall are presented in Table 5. It can be observed that there is considerable improvement in all the performance evaluation metrics. It shows the requirement of enhancement.

The image dataset was updated by the fusion of images enhanced using power law transformation and log transformation. Optimal values set after performing detailed experiments were used for the fusion of input images as described in Section 3. Modified CNN models were used for the extraction of features and classifications. Classification results are shown in Table 6 and the confusion matrix of Mobilenetv2 is shown in Table 7 to enable a better understanding of the results. There is a drastic increase in accuracy of the proposed technique and performance improvement can be observed, from 58.82% prior to enhancement to 96.71% using the best performing classifier MobileNetV2.

In Table 8, a sample of images are shown which are accurately as well as poorly classified by the Mobilenetv2 model for better analysis. The performance of our suggested model is not up to the mark against the polyp, dyed-lifted polyps and ulcerative colitis class as shown in Table 7.

In order to further validate the performance of the proposed model [16,17,18,19,20,21] we have compared the results with existing techniques. Our proposed system based on simple preprocessing and using a lightweight CNN model has outperformed the existing techniques. Results presented in Table 9 clearly show the better performance in terms of accuracy. However, our system is consistent in terms of accuracy, precision, and recall.

## 6. Conclusions

Over the period of the last two decades, an increase is reported for gastrointestinal diseases. Complications may occur if these diseases are not addressed at an early stage. Early detection can be made possible if computer vision-based automated systems are utilized. A major challenge with these automated approaches is the poor contrast and improper illumination of images used for the detection and classification of diseases. In this paper, I have addressed this issue by the employment of automated detection and classification of ulcers by devising a contrast and illumination invariant technique. Results on the WCE Kvasir dataset show that not only an improvement in the classification accuracy but also an improvement in the convergence of deep learning models was achieved. Results show that I managed to achieve a 96.71% accuracy which is an improvement over the existing techniques. In future work, different contrast and illumination techniques could be explored as well as multiple different measures could be applied for checking the quality of images.

## Figures and Tables

**Table 1:** Results after applying power law transformation.

Power Law Transformation	Before Processing	After Processing	Mean Squared Error
$\alpha =1.5*P^{0.7}$	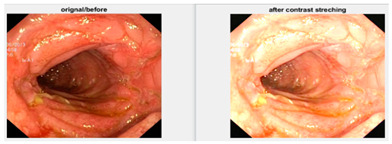		343.92
$\alpha =1.9*\;P^{0.8}$	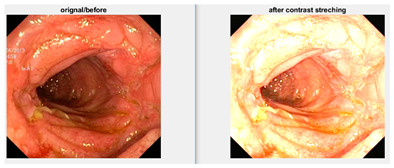		389.47
$\alpha =1.5*\;P^{0.9}$	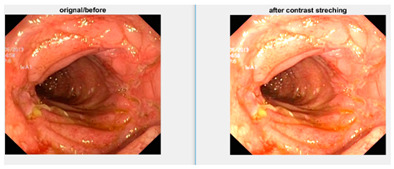		219.64
$\alpha =1.5*\;P^{1}$	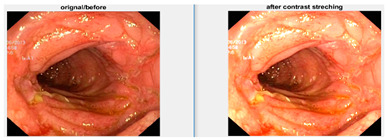		251.17

**Table 2:** Results after applying Log Transformation.

Log Transformation	Before Processing	After Processing	Mean Squared Error
α=2.5∗ Log (P+1)	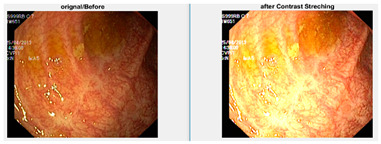		351.44
α=2.3∗ Log (P+1)	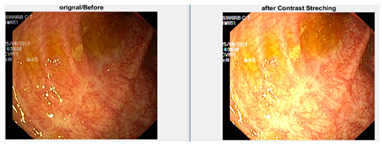		397.13
α=2∗ Log (P+1)	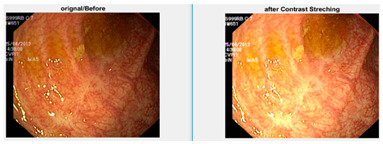		229.08
α=1.9∗ Log (P+1)	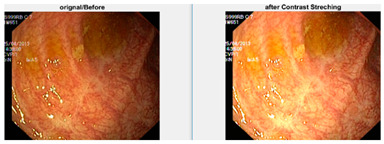		268.32

**Table 3:** CNN models classification accuracy, precision, and recall (%) prior to enhancement.

Model	Accuracy	Precision	Recall
Mobilenetv2	**58.82**	**57.32**	**59.35**
VGG19	54.71	48.67	51.39
VGG16	57.65	56.45	54.78
ResNet50	52.35	51.45	49.45
ResNet101	33.53	38.45	41.34

**Table 4:** Classification, precision, and recall (%) on enhanced dataset using power law transformation.

Model	Accuracy	Precision	Recall
Mobilenetv2	**73.71**	**83.78**	**79.87**
VGG19	64.23	57.98	78.98
VGG16	56.38	67.97	56.87
ResNet50	67.98	73.87	76.78
ResNet101	56.78	51.86	53.87

**Table 5:** Classification, precision, and recall (%) on enhanced dataset using log transformation.

Model	Accuracy	Precision	Recall
Mobilenetv2	**77.71**	**88.00**	**87.00**
VGG19	67.54	59.45	45.87
VGG16	51.78	56.97	46.87
ResNet50	44.85	54.87	41.78
ResNet101	67.43	58.86	56.87

**Table 6:** Proposed technique classification performance (%).

Model	Accuracy	Precision	Recall
Mobilenetv2	**96.71**	**97.22**	**93.21**
VGG19	94.22	96.64	91.65
VGG16	52.38	64.97	59.87
ResNet50	34.85	43.87	45.78
ResNet101	43.43	57.86	67.87

**Table 7:** Confusion matrix of our proposed model (Mobilenetv2).

	Detected Class (%)								
Actual Class (%)		Dyed-lifted polyps	Dyed resection margins	Esophagitis	Normal cecum	Normal pylorus	Normal z-line	Polyps	Ulcerative colitis
	Dyed-lifted polyps	**92**	4	2	0	0	0	1	1
	Dyed resection margins	1	**94**	2	0	0	0	3	0
	Esophagitis	0	0	**97**	0	0	0	1	2
	Normal cecum	0	0	1	**98**	0	0	0	1
	Normal pylorus	0	0	1	2	**94**	2	1	0
	Normal z-line	0	1	0	1	1	**96**	0	1
	Polyps	3	2	0	0	0	0	**92**	1
	Ulcerative colitis	0	2	2	0	0	0	1	**95**

**Table 8:** Sample of classified images by Mobilenetv2.

Sr.	Images	Class	Finding
1	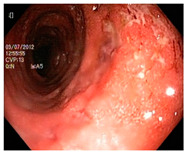	Esophagitis	Poorly classified
2	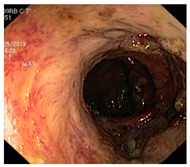	Ulcerative-colitis	Poorly classified
3	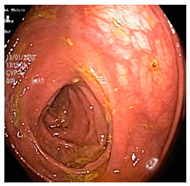	Normal cecum	Accurately classified
4	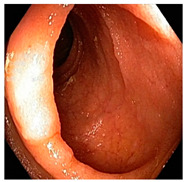	Normal pylorus	Accurately classified

**Table 9:** Comparison of proposed technique with state-of-the-art techniques.

Year	Description	Performance
2018 [4]	AlexNet based CNN model with different preprocessing steps such as data augmentation and enhancement.	95.16%
2017 [9]	GDPNet model	88.9%
2019 [10]	Logistic regression	82.6%
	Ridge regression	83.3%
2018 [11]	Modern CAD which contains handcrafted features, fusion features, and deep CNN features.	96.5%
2017 [19]	Model based on pre-trained VGGNet, ResNet, and inception with different preprocessing steps.	77.1%
2022 [21]	An adaptive aggressive features module was utilized to obtain deep discriminated features.	96.37%
Proposed model		**96.71%**

## Data Availability

This study did not report any data.
